# Exploring the Research Progress of Vascular Dementia and Key Regulatory Molecules: E2F1

**DOI:** 10.3390/ijms27094008

**Published:** 2026-04-30

**Authors:** Fengwei Zhang, Zhihua Hao, Mingrong Song, Zihan Zhao, Wanying Li, Jing Chen

**Affiliations:** Key Laboratory of Chinese Medicine Basic Theory Research in Heilongjiang Province, College of Basic Medicine, Heilongjiang University of Chinese Medicine, Harbin 150040, China; zhangfengwei2009@126.com (F.Z.); haozz162@163.com (Z.H.); songmingrong3718@163.com (M.S.); zzh02134213@163.com (Z.Z.); liwanying20027@163.com (W.L.)

**Keywords:** vascular dementia (VaD), pathogenesis, E2F1, therapeutic target

## Abstract

Vascular dementia (VaD) is a type of dementia caused by cerebrovascular factors, which can arise from both ischemic cerebrovascular disease and hemorrhagic cerebrovascular disease. The incidence rate of VaD is second only to Alzheimer’s disease and dementia with Lewy bodies. Currently, there are no effective drugs specifically targeting VaD, making the discovery of new therapeutic targets of great significance. This article provides an overview of the research progress on VaD, with a focus on elucidating its pathogenesis, aiming to identify targets that play a regulatory role in the mechanism. Finally, our attention is drawn to the transcription factor E2F1. Through research, it has been found that E2F1 is involved in biological processes such as cell cycle regulation and apoptosis and plays a certain role in neurodegenerative diseases and ischemic encephalopathy. It also participates in the pathogenesis of vascular dementia, suggesting that E2F1 is a key regulatory molecule for VaD and may become a potential pharmacological therapeutic target, which warrants further in-depth research.

## 1. Introduction

Vascular dementia (VaD) is a type of dementia caused by cerebrovascular factors, which can be induced by ischemic cerebrovascular disease and hemorrhagic cerebrovascular disease [1]. Its clinical manifestations include memory impairment; impaired attention, information processing ability, and executive function; early gait disorders; and depression, apathy, and personality changes [2,3]. According to estimates by the World Health Organization, approximately 82 million people will suffer from dementia by 2050 [4]. Among all subtypes of dementia, vascular dementia accounts for about 20% of the total prevalence of dementia [5], second only to Alzheimer’s disease and dementia with Lewy bodies [6]. The average age at onset, diagnosis, and death for vascular dementia is 67.5 ± 7.2 years, 73.5 ± 7.0 years, and 77.0 ± 6.9 years, respectively. The survival time after diagnosis (3.2 ± 1.4 years) is shorter than that of common dementias such as Alzheimer’s disease (5.8 ± 2.0 years) and dementia with Lewy bodies (4.7 ± 1.8 years) [7]. Vascular dementia is associated with multiple risk factors, and the risk factors for cardiovascular and cerebrovascular diseases are also risk factors for vascular dementia, mainly including poor dietary habits [8], obesity [9], hypertension [10], hypercholesterolemia [11], excessive alcohol consumption [12], diabetes [13], smoking [14], and lack of exercise [15]. Vascular dementia is mainly divided into four subtypes: post-stroke dementia, subcortical ischemic vascular dementia, multi-infarct dementia, and mixed dementia [16]. This is why the pathogenesis of vascular dementia is complex, but the main pathogenesis of different vascular dementia subtypes is common [17]. The current drug treatment for vascular dementia mainly focuses on controlling vascular risk factors, improving cerebral blood flow and neuroprotection. The clinical medications mainly include cholinesterase inhibitors such as donepezil and galantamine, as well as the N-methyl-D-aspartate receptor (NMDAR) antagonist memantine. These drugs were originally used for Alzheimer’s disease. Although they are now also used for the treatment of vascular dementia, they only have a mild improvement effect on specific types of vascular dementia, such as mixed dementia, and are not sufficient to support their widespread use in patients with vascular dementia [18]. The role of epigenetic regulation and transcriptional factors is gradually being emphasized. Research in this area may help identify potential pharmacological therapeutic targets for vascular dementia. This review proposes that the transcription factor E2F1, as a multi-dimensional regulatory molecule, may become a promising new pharmacological therapeutic target for vascular dementia.

## 2. The Etiology, Pathology, and Pathogenesis of VaD

### 2.1. Etiology and Pathology

The primary etiology of vascular dementia is neuronal injury resulting from chronic cerebral hypoperfusion induced by vascular lesions. Vascular lesions mainly refer to large vessel diseases such as carotid stenosis, as well as small vessel diseases, including small (micro) infarcts, microbleeds, cerebral amyloid angiopathy (CAA), arteriosclerosis, and genetic cerebral arterial diseases such as Notch 3 gene defects [19,20,21]. The brain parenchyma is supplied with blood by perforating arteries, which originate from the external cerebral arteries and form a complex anastomotic network of blood supply after penetrating the brain. Insufficient blood supply from the external cerebral arteries can lead to decreased blood flow in the perforating arteries, which results in white matter lesions and gray matter neuronal damage [22,23]. The spaces surrounding the perforating arteries, called Virchow-Robin spaces (abbreviated as VRS), serve as lymphatic drainage channels in the brain [24,25,26]. The pathological manifestation of VRS in small-vessel diseases is widening, which can lead to lymphatic clearance dysfunction and cause nerve cell damage [27,28,29].

### 2.2. Pathogenesis

#### 2.2.1. Excitotoxicity

Chronic cerebral hypoperfusion can induce excitotoxicity in the central nervous system, which is one of the pathogeneses of vascular dementia [30,31,32]. Excitotoxicity refers to the death of neuronal cells caused by the continuous activation of excitatory amino acid receptors. Glutamate is the predominant excitatory amino acid in the brain. Ischemia and hypoxia lead to the continuous release of glutamate from presynaptic neurons, which excessively activates postsynaptic excitatory amino acid receptors such as N-methyl-D-aspartate receptors (NMDAR), causing a large influx of calcium ions and resulting in intracellular calcium overload, ultimately leading to cell death [33].

In addition, other cells in the brain also participate in the excitotoxic process. Glutamate receptors on endothelial cells are continuously activated, leading to a large influx of calcium ions into the endothelial cells. This, in turn, disrupts mitochondrial function, generates reactive oxygen species, and damages cell metabolism, ultimately disrupting the integrity of the blood–brain barrier and causing neurotoxic damage [34,35]. Glutamate transporters on astrocytes transport a large amount of extracellular glutamate into astrocytes, where it is converted into glutamine by glutamine synthetase and then recycled into presynaptic neurons. Glutamate transporters are energy-dependent and transport glutamate along the sodium ion concentration gradient. During ischemia and hypoxia, energy supply is blocked, and coupled with strong depolarization, intracellular sodium ion concentration rises sharply, leading to the reverse transport of glutamate from the intracellular to the extracellular along the sodium ion concentration gradient, resulting in a large accumulation of glutamate and exacerbating excitotoxicity [36,37,38]. Chronic cerebral hypoperfusion can activate microglia, which transport cysteine into the cells through the xc^−^ antiporter and simultaneously transport glutamate out of the cells in a 1:1 ratio. Moreover, activated microglia also release relevant factors that impair the function of excitatory amino acid transporters in other cells, further aggravating the accumulation of extracellular glutamate and excitotoxic damage [39] (Table 1).

#### 2.2.2. Oxidative Stress

Oxidative stress is the core mechanism underlying the occurrence and development of vascular dementia. It does not act independently but is intertwined with inflammatory reactions, mitochondrial damage, and ferroptosis, forming a complex network that leads to nervous system damage. Oxidative stress refers to a pathological state in which the production of reactive oxygen species (ROS) and reactive nitrogen species (RNS) in the body exceeds the endogenous antioxidant defense capacity, resulting in oxidative damage to cellular structures such as lipids, proteins, and DNA. ROS include superoxide anion (O_2_^−^), hydrogen peroxide (H_2_O_2_), hydroxyl radical (·OH), etc. Under normal physiological conditions, ROS participates in cell signaling, but excessive amounts trigger toxic reactions [40,41,42,43].

Cerebral ischemia–reperfusion injury explosively produces ROS through pathways such as mitochondrial electron transport chain leakage [44,45,46]. Nicotinamide adenine dinucleotide phosphate (NADPH) oxidase (NOX) on the cell membrane is another important source of ROS [47,48,49]. Cyclooxygenase (COX) can increase the production of ROS in endothelial cells through inflammatory reactions [50]. These three are the main sources of oxidative stress in the vascular system [51]. In addition, after chronic cerebral ischemia, heme decomposition releases free iron, which generates highly toxic hydroxyl radicals (·OH) through the Fenton reaction (Fe^2+^ + H_2_O_2_ → Fe^3+^ + ·OH + OH^−^) [52]. The decrease in the activity of antioxidant enzymes such as superoxide dismutase (SOD) and glutathione peroxidase (GPx), as well as the imbalance of the antioxidant system caused by glutathione (GSH) depletion, will aggravate the excessive accumulation of ROS [53,54,55]. These are the mechanisms underlying the generation of oxidative stress in vascular dementia.

The role of oxidative stress in the pathogenesis of vascular dementia is promoted through multiple pathways in a synergistic manner. ROS inhibits the bioavailability of nitric oxide (NO), leading to vascular dilation disorders and abnormal cerebral blood flow regulation [56,57]. ROS activates matrix metalloproteinases (MMPs), disrupts the basement membrane of cerebral vascular endothelial cells, and leads to blood–brain barrier leakage [58]. Oxidation causes lipid peroxidation, protein carbonylation, and DNA fragmentation, induces mitochondrial apoptosis pathways, and results in neuronal death or synaptic dysfunction [42,59,60,61]. ROS attacks oligodendrocytes, leading to demyelination, white matter damage, decreased cognitive conduction velocity, and impaired executive function [62]. ROS continuously activates microglia to release pro-inflammatory cytokines, forming a positive feedback loop of “oxidation-inflammation” and damaging neurons [63].

Oxidative stress is both an initiator and an amplifier in the pathogenesis of vascular dementia, driving cognitive decline through multiple dimensions such as direct damage to blood vessels, neurons, and neuroglial cells, exacerbating inflammation, and disrupting the blood–brain barrier.

#### 2.2.3. Neuroinflammation

Neuroinflammation plays a pivotal role in the pathogenesis of vascular dementia. Chronic cerebral hypoperfusion not only directly induces energy metabolism disorders and ischemic injury but also activates the innate immune response and initiates a neuroinflammatory cascade by inducing hypoxia and stress signals [64,65]. Hypoxia-inducible factors (HIFs) are core regulators of hypoxic adaptation. In myeloid cells (such as macrophages or neutrophils), HIFs not only regulate glycolysis and innate immunity but also directly promote the expression of pro-inflammatory genes and cell migration, amplifying the inflammatory response [66]. Furthermore, the disruption of the blood–brain barrier (BBB) caused by chronic cerebral hypoperfusion allows peripheral immune cells (such as monocytes, T cells) to infiltrate the brain parenchyma, further exacerbating local inflammation [67].

In the inflammatory microenvironment induced by chronic cerebral hypoperfusion, pro-inflammatory cytokines such as interleukin-6 (IL-6), tumor necrosis factor alpha (TNF-α), and interleukin-1β (IL-1β), as well as chemokines such as monocyte chemoattractant protein-1 (MCP-1), are key effector molecules. IL-6 exacerbates vascular damage by promoting the formation of atherosclerotic plaques and inducing the expression of endothelial cell adhesion molecules such as intercellular adhesion molecule-1 (ICAM-1). Additionally, IL-6 can activate microglia and promote the release of pro-inflammatory cytokines, forming a vicious cycle of “inflammation-vascular damage” [68]. Patients with vascular dementia exhibit elevated levels of TNF-α in both blood and cerebrospinal fluid (CSF), which is positively correlated with the severity of white matter hyperintensities (WMH) [69]. TNF-α induces the expression of inducible nitric oxide synthase (iNOS) and cyclooxygenase-2 (COX-2) by activating the NF-κB pathway, increasing the production of nitric oxide (NO) and prostaglandin E2 (PGE2) and directly leading to oxidative damage to neurons [70]. IL-6 and TNF-α can also damage vascular endothelial cells and reduce the expression of claudin-5, a tight junction protein of the BBB, leading to plasma protein leakage, inducing brain edema and white matter ischemia [69,71]. After chronic cerebral hypoperfusion, the expression of MCP-1 in vascular endothelial cells is upregulated, recruiting peripheral monocytes to infiltrate the brain parenchyma through C-C chemokine receptor 2 (CCR2), differentiate into pro-inflammatory macrophages, and release ROS and proteases such as matrix metalloproteinase 9 (MMP-9), thereby damaging the blood–brain barrier (BBB) [72].

The complement system, as a core component of innate immunity, interacts with the coagulation system in vascular dementia, exacerbating inflammatory damage [73]. Studies have found that in chronic cerebral hypoperfusion models, the deposition of C5 complement protein in the white matter region of the corpus callosum significantly increases, consistent with the temporal progression of white matter damage. C5-deficient mice exhibit significantly reduced white matter ischemic damage and reactive glial cell proliferation, suggesting that C5 modulates the activation of the NLR family pyrin domain-containing 3 (NLRP3) inflammasome, thereby promoting the release of pro-inflammatory cytokines, including IL-1β, mediating white matter damage [74,75]. Furthermore, the synergistic action of complement and coagulation factors can enhance the permeability of endothelial cells, further damaging the integrity of the BBB [73].

Matrix metalloproteinases (MMPs), especially MMP-3 and MMP-9, play a dual role in vascular remodeling, but their abnormal activation in vascular dementia can damage the BBB and lead to myelin damage. In the brain tissue of patients with Binswanger disease (a specific subtype of VaD), MMP-3 is continuously expressed in areas infiltrated by microglia/macrophages, suggesting its involvement in white matter demyelination and axonal degeneration processes. MMPs promote inflammatory cell infiltration by degrading the extracellular matrix and form a positive feedback loop with pro-inflammatory cytokines such as TNF-α, accelerating the progression of VaD [76]. Additionally, MMPs inhibit myelin regeneration by degrading the oligodendrocyte precursor cell survival factor, platelet-derived growth factor [77].

Microglia, the innate immune cells in the brain, exhibit biphasic functions in VaD. Early activation allows them to clear necrotic debris, but sustained activation shifts to a pro-inflammatory M1 phenotype, releasing toxic molecules such as IL-6, TNF-α, and iNOS, exacerbating white matter damage [78]. Chronic cerebral hypoperfusion can activate NLRP3 and melanoma differentiation-associated protein 2 (AIM2), triggering cysteinyl aspartate specific proteinase-1 (caspase-1)-dependent maturation and release of IL-1β, inducing microglia polarization to the M1 phenotype, promoting the generation of ROS and NO, and directly damaging oligodendrocytes and neurons; meanwhile, IL-1β can also enhance the expression of adhesion molecules on endothelial cells, recruit more immune cells for infiltration, and form a vicious cycle of “inflammasome-pro-inflammatory cytokines-cell infiltration” [79]. Chronic cerebral hypoperfusion can activate microglia through the p38 mitogen-activated protein kinase (p38 MAPK) pathway, enhancing the expression of pro-inflammatory cytokines and leading to damage to white matter and hippocampal neurons [80,81]. Furthermore, microglia can secrete MMP-9 to directly degrade myelin basic protein (MBP), resulting in demyelination and further disruption of neural conduction [76].

In summary, inflammation is a key link in the pathogenesis of VaD, permeating the entire process of chronic cerebral hypoperfusion, white matter damage, and neuronal loss, involving multidimensional regulation by pro-inflammatory cytokines, adhesion molecules, complement, matrix metalloproteinases, inflammasomes, and microglia.

#### 2.2.4. BBB Disruption

The blood–brain barrier (BBB), as a crucial interface between the central nervous system (CNS) and peripheral circulation, plays a significant role in the pathogenesis of vascular dementia (VaD) by regulating substance exchange and maintaining brain homeostasis. Studies have shown that BBB disruption is not only a pathological feature of VaD but also a factor contributing to disease progression. The BBB is composed of brain microvascular endothelial cells (BMECs), astrocyte end feet, pericytes, and the basement membrane (BM) [82]. Its core functions include: strictly restricting harmful substances such as pathogens, toxins, and large molecular proteins from entering the brain parenchyma; regulating the exchange of nutrients and metabolic waste through transportation mediated by glucose transporters (GLUT) and receptors such as low-density lipoprotein receptor-related protein 1 (LRP1); maintaining the stability of ions and neurotransmitters within the brain [83].

Unlike peripheral vascular endothelial cells, BMECs exhibit three unique characteristics: they express tight junction proteins (TJs), such as claudin-5, occludin, and zona occludens-1 (ZO-1), to seal the paracellular pathway; they lack fenestrations and have extremely low pinocytosis; and they highly express efflux transporters to prevent exogenous substances from entering the brain [58]. Pericytes, embedded between BMECs and astrocyte end feet, which regulate BBB-specific gene expression in BMECs and induce polarization of astrocyte end-feet, are key cells in the formation and stability of the BBB [84]. Astrocyte end feet encase blood vessels and maintain BBB integrity by secreting paracrine factors such as laminin [85].

Chronic cerebral hypoperfusion or ischemic reperfusion injury can lead to BBB cell damage or pro-inflammatory changes, subsequently causing BBB disruption. In patients with VaD and animal models, BMECs exhibit reduced mitochondrial content, increased pinocytic vesicles, and downregulated GLUT1 expression, leading to insufficient brain energy supply [86]. A significant decrease in the number of pericytes results in loss of vascular stability [84,87]. Pericyte degeneration can also induce white matter microcirculation disorders, manifested as fibrinogen deposition, reduced blood flow, and myelin loss [84]. The level of the soluble marker platelet-derived growth factor receptor β (PDGFRβ), reflecting pericyte damage, is directly correlated with cognitive impairment [88]. Astrocytes are activated, leading to reduced vascular coverage, upregulation of glial fibrillary acidic protein (GFAP), and downregulation of aquaporin 4 (AQP4), which damages the clearance ability of cerebral interstitial fluid [89]. The level of lipocalin-2 (LCN2) secreted by reactive astrocytes increases significantly, mediating the death of hippocampal neurons [90].

The disruption of the BBB and cognitive impairment in vascular dementia (VaD) form a “vicious cycle”: increased BBB permeability leads to the entry of blood-borne toxins into the brain parenchyma, activating glial cells and neurons, triggering oxidative stress and cell death; neuronal death further weakens brain metabolic demand, leading to abnormal regulation of cerebral blood flow and exacerbating cerebral hypoperfusion; cerebral hypoperfusion, in turn, intensifies BBB disruption, forming a positive feedback loop [91]. In summary, BBB disruption is an important link in the pathogenesis of VaD, involving multiple pathways such as BBB cell damage or inflammatory changes, oxidative stress, and toxic factor mediation (Table 2).

In summary, the core pathogenesis of VaD includes excitotoxicity, oxidative stress injury, neuroinflammation, and BBB disruption. In the treatment of VaD, traditional studies have mainly focused on the association between vascular damage and neurodegeneration. The effectiveness of drugs developed based on this is limited, and thus the role of epigenetic regulation and transcriptional factors has gradually gained attention. E2F1 is one of the members of the E2F family. As a core transcription factor for cell cycle regulation, it mainly regulates the transition from the G1 phase to S phase of cells and is also involved in biological processes such as DNA damage repair and apoptosis [92]. Recent studies have found that E2F1 exacerbates nerve damage through multiple pathways and participates in the nerve-damage process of VaD by regulating cell apoptosis, autophagy, inflammatory response, oxidative stress, and angiogenesis, and is expected to become a potential pharmacological therapeutic target.

## 3. Characteristics of E2F1

The adenovirus E2 promoter binding factor (E2F) [93,94], a transcription factor family encoded by eight genes and containing eight subtypes (E2F1-8), is regulated by alternative splicing or different transcription start points, resulting in protein isoforms that share a common DNA-binding domain [95,96,97,98,99]. Among them, E2F1 is the earliest discovered E2F transcription factor, regulating the expression of cell cycle-related genes including DNA repair [100,101,102,103,104,105], cell proliferation, and apoptosis [106,107]. Therefore, it is one of the most important proteins for cells [108,109,110]. The regulatory process of E2F1 on the expression of various proteins occurs in the cell nucleus [110]; however, in the cytoplasm, E2F1 can interact directly with mitochondria in a non-transcriptional manner [111,112,113]. E2F1 needs to bind to dimerization proteins (DP) or retinoblastoma proteins (pRB) [114] to exert activity in the promoter region of genes. The binding of E2F1 to pRB inhibits the formation of the E2F1-DP complex, blocks the recruitment of transcriptional coactivators to the gene promoter region, and affects the expression of cell cycle-related proteins [115]. When pRB is phosphorylated by cyclin-dependent kinases (CDKs), E2F1 is released and can promote the expression of cell cycle-related genes [116,117,118,119]. Genome-wide localization studies have shown that, in addition to its key role in cell cycle regulation, E2F1 also regulates numerous cellular pathways by binding to hundreds of gene promoter regions [120,121,122,123]. E2F1 is a transcription factor that plays a crucial role in cell fate and is capable of regulating gene expression related to DNA replication, DNA damage repair, cell cycle, and programmed cell death [124,125].

## 4. Expression of E2F1 in the Cell Cycle

E2F1 is involved in regulating the expression of cell cycle-related genes [126]. The E2F1 gene is induced to express in the late G1 phase, activating the inhibitory factors in the E2F family during the S phase. As the level of inhibitory factors increases, the transcription of E2F1 is inhibited, and the E2F program in the G2 phase is shut down [127,128,129,130,131]. E2F1 can undergo post-translational modifications such as phosphorylation, methylation, ubiquitination, and acetylation to recognize and bind regulatory elements, influencing RNA polymerase recognition and binding to promoters, thus regulating transcription [132,133,134,135,136]. The degradation of E2F1 is associated with the ubiquitin ligase late-stage promoting complex (APC/C) and is considered to be an APC/C-dependent ubiquitin-targeted process [137,138]. The expression of E2F1-driven APC/C inhibitors inhibits the activity of APC/C. By late S phase, E2F7 or E2F8 inhibits the expression of APC/C inhibitors, and E2F1 binds to APC/C and is degraded [139,140]. However, recent studies have suggested that members of the F-box protein family, such as cyclin F (CCNF), promote the degradation of E2F1 at the S post-midpoint stage in order to prevent the initiation of inappropriate DNA replication programs and to avoid disrupting the normal cell cycle progression [141].

## 5. E2F1 and Apoptosis

**Figure 1:**
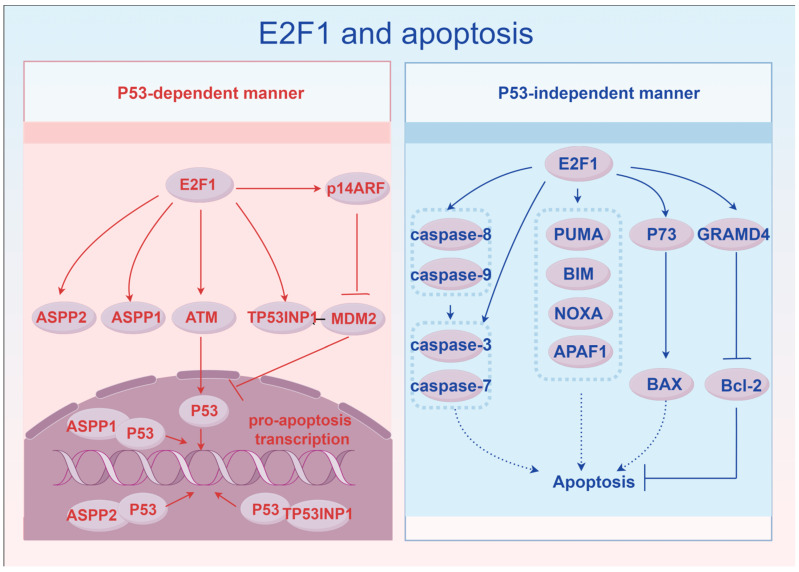
E2F1 and apoptosis. By Figdraw. It was proposed that E2F1 promotes apoptosis in two ways: P53-dependent and P53-independent. The signaling pathways by which E2F1 regulates apoptosis were introduced in the two methods. 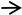 represents direct enhancement, 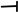 represents direct inhibition, and 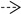 represents indirect enhancement. ASPP1, ASPP2, and TP53INP1 are tran-scriptional co-activators that bind to P53 and enhance its transcriptional function.

E2F1 promotes cell apoptosis in two ways. Firstly, in a P53-dependent manner, E2F1 can activate the transcription of p14ARF, an inhibitor of mouse double microbody gene 2 protein (MDM2), which in turn inhibits the pro-apoptotic function of P53 [142,143]. E2F1 can also promote the transcription of genes such as ataxia telangiectasia mutated (ATM), which activates P53 through phosphorylation [144,145,146,147]. Additionally, E2F1 can activate the transcription of P53 co-activators such as P53 apoptosis-stimulating protein 1 (ASPP1), P53 apoptosis-stimulating protein 2 (ASPP2), and tumor protein P53-induced nuclear protein 1 (TP53INP1), thereby enabling P53 to exert its pro-apoptotic function [148,149,150,151]. Secondly, in a P53-independent manner, E2F1 can directly activate the transcription of genes such as p53 upregulated modulator of apoptosis (PUMA), Bcl-2 interacting mediator of cell death (BIM), phorbol 12-myristate 13-acetate-induced protein 1 (NOXA), apoptotic protease activating factor 1 (APAF-1), cysteinyl aspartate specific proteinase-3, -7, -8 and -9 (caspase-3, -7, -8 and -9), thereby exerting a pro-apoptotic effect [152,153,154,155,156]. E2F1 can also activate the transcription of P73 [157], and P73 can activate the Bcl-2-associated X protein (BAX), exerting a pro-apoptotic effect [158]. P73 can also activate the GRAM domain-containing protein 4 (GRAMD4), which is located on the mitochondria and can inhibit B-cell lymphoma-2 (Bcl-2) on its membrane, thereby exerting a pro-apoptotic effect [159]. Furthermore, when DNA damage occurs and nucleolar stress is induced, p14ARF in the nucleolus transfers to the nucleoplasm, recruiting E2F1 from the nucleoplasm to the nucleolus [160,161]. Ribosomal RNA processing protein 1 homolog B (RRP1B) in the nucleolus binds to the DNA-binding domain of E2F1, enhancing the transcription of pro-apoptotic genes such as caspase-3 and -7 [162] (Figure 1).

## 6. The Role of E2F1 in Neurodegenerative Diseases

**Figure 2:**
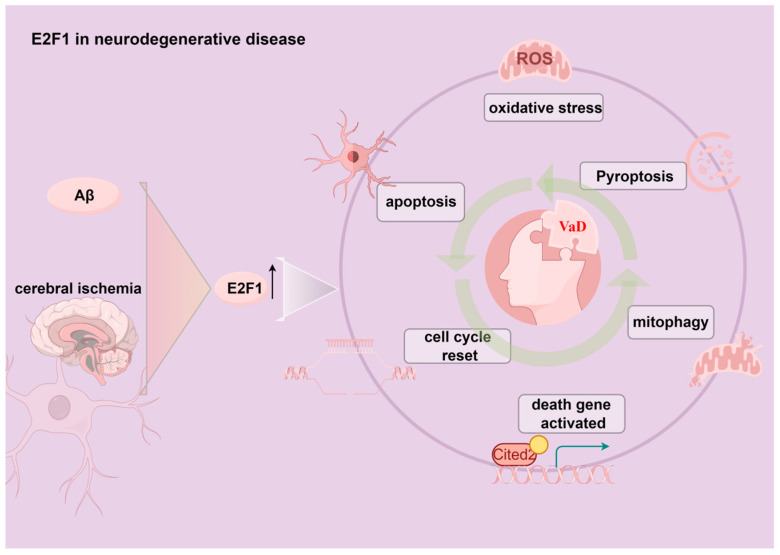
E2F1 in neurodegenerative disease. By Figdraw. Aβ and cerebral ischemia induce an upregulation of E2F1 levels, leading to a series of events such as oxidative stress, pyroptosis, mitophagy, activation of death genes like Cited2, cell cycle reset, apoptosis, and ultimately triggering VaD.

When cell cycle progression is disrupted or arrested, E2F1 can drive the apoptosis response of neurons [163]. E2F1 is highly expressed in the hippocampal tissue of AD model rats [164], and its high expression is a major factor contributing to cognitive impairment after traumatic brain injury [165], indicating that E2F1 is a risk factor for dementia. Furthermore, it has been reported that E2F1 participates in regulating apoptosis events induced by oxidative stress [166], and downregulation of E2F1 can inhibit cell pyroptosis caused by oxidative stress [167]. Moreover, knockdown of E2F1 can reduce ROS levels and mitigate mitochondrial autophagy damage by inhibiting triggering receptor expressed on myeloid cells 1 (TREM1), and silencing E2F1 can effectively reduce mitochondrial damage and improve neurological function in mice with cerebral ischemia–reperfusion injury [168]. In addition, E2F1 can activate the Cbp/p300-interacting transactivator, with Glu/Asp-rich carboxy-terminal domain 2 (Cited2), and promote neuronal cell death [169], and AD pathogenic protein Aβ can induce neuronal death by upregulating E2F1 [170]. This suggests that E2F1 may damage neurons and trigger dementia by inducing oxidative stress and mitochondrial dysfunction (Figure 2).

## 7. The Role of E2F1 in Ischemic Brain Injury

Within the penumbra region caused by photothrombotic stroke (PTS), the levels of E2F1 and p53 are significantly elevated [171]. This phenomenon aligns with the roles of E2F1 [172] and p53 [173] in the tissue response of the penumbra after transient ischemic stroke in rats. Blocking the E2F1/p53 signaling axis can inhibit the process of neuronal apoptosis [174]. On the other hand, E2F1 can exacerbate oxidative stress-triggered apoptosis through Apoptosis Signal-regulating Kinase 1 (ASK1)/c-Jun N-terminal kinase (JNK) cascade [175,176]. As a crucial driver of stress-induced apoptosis, JNK shows an elevated trend in the penumbra region caused by PTS [171] and can enhance the pro-apoptotic effect of p53 [177]. Furthermore, p38MAPK, which is involved in regulating the expression of E2F1 and p53, is also upregulated in the penumbra region after PTS. Its role in enhancing E2F1 expression may be achieved through mitogen-activated protein kinase-activated protein kinase 2 (MAPKAPK2) [178,179], which also exhibits a high expression level in this region. This suggests that E2F1 is closely related to the mechanism of ischemic brain tissue damage. Therefore, it is inferred that E2F1 can be further studied as a key regulatory molecule for vascular dementia.

## 8. Involvement of E2F1 in the Pathogenesis of VaD

**Figure 3:**
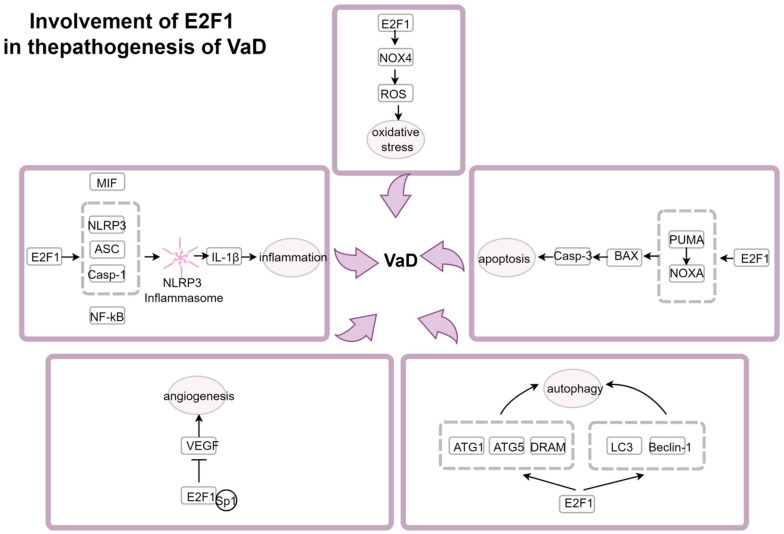
Involvement of E2F1 in the pathogenesis of VaD. By Figdraw. The signal pathways involved in the regulation of E2F1 in five aspects, namely promoting apoptosis, interfering with autophagy, acti-vating inflammation, regulating oxidative stress and inhibiting angiogenesis, were proposed, in-dicating the specific mechanism by which E2F1 is involved in the neural injury process of VaD. 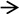 represents enhancement, 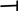 represents inhibition.

E2F1 regulates neuronal apoptosis: E2F1 can promote the transcription of PUMA and NOXA, the initiators of the apoptotic process, further activating the downstream BAX and activating Caspase-3, thereby promoting the process of apoptosis [180,181]. E2F1 disrupts autophagic homeostasis and exacerbates neural damage: Under conditions of oxidative stress or nutrient deficiency, E2F1 promotes the expression of microtubule-associated protein 1 light chain 3 (LC3) and Beclin-1, regulating autophagy in cells [182]. The activation of E2F1 also upregulates the expression of autophagy-related gene-1 (ATG1), ATG5, and damage-regulated autophagy modulator (DRAM), enhancing autophagy [183]. E2F1 activates neuroinflammation: E2F1 upregulates the expression of NLRP3, apoptosis-associated speck-like protein containing a CARD (ASC), and caspase-1, promoting the assembly of NLRP3 inflammasomes and the maturation and release of IL-1β [184]. E2F1 can also promote the generation of NLRP3 inflammasomes by activating the NF-κB pathway [185]. Additionally, E2F1 can promote the generation of NLRP3 by targeting the transcription of its subunit, macrophage migration inhibitory factor (MIF) [186]. E2F1 regulates oxidative stress damage: One binding site of E2F1 can bind to the promoter of nicotinamide adenine dinucleotide phosphate (NADPH) oxidase 4 (NOX4), promoting the transcription of NOX4 and the production of downstream ROS [187], indicating that E2F1 is involved in the mechanism of oxidative stress damage. E2F1 inhibits angiogenesis and collateral circulation compensation: Moderate angiogenesis can increase blood flow supply to ischemic areas and improve the prognosis of VaD. However, studies [188] have shown that E2F1 competitively binds to the specificity protein 1 (Sp1) binding site on the promoter of vascular endothelial growth factor (VEGF), inhibiting its transcription and angiogenesis. In conclusion, E2F1 plays a regulatory role in the core pathogenesis of VaD, providing a fundamental basis for further research on E2F1 as a pharmacological therapeutic target for VaD (Figure 3).

## 9. Conclusions and Prospects

This article provides a comprehensive review of the current research status of vascular dementia. As effective drugs for vascular dementia have not yet been developed, the discussion on drug aspects is not included in this review. Instead, this article mainly focuses on the pathogenesis of vascular dementia, aiming to identify potential molecules that may play a regulatory role in the mechanism, thereby providing a direction for finding new pharmacological therapeutic targets. The role of transcription factor E2F1 in the cell cycle, especially in apoptosis, has caught our attention. Simultaneously, we have found that E2F1 has a certain regulatory effect on the occurrence and progression of neurodegenerative diseases and ischemic brain diseases, suggesting that E2F1 is likely to play a regulatory role in the occurrence and development of vascular dementia. Further research has revealed that E2F1 participates in the neural injury process of VaD by promoting apoptosis, interfering with autophagy, activating inflammation, regulating oxidative stress and inhibiting angiogenesis, and is a key regulatory molecule for VaD. It is worth noting that this study is at an early stage of pharmacological therapeutic target research, and the conclusions are based on animal or cell models of VaD. The purpose is to provide a promising exploration direction for the development of pharmacological therapeutic targets for VaD.

Future research can further explore E2F1 as a pharmacological therapeutic target for VaD, mainly considering its safety and efficacy. For instance, how to specifically reduce the overexpressed E2F1 in the brain tissue cells of VaD, and how to act on E2F1 in VaD brain tissue through a specific delivery system without being restricted by the BBB, etc. It is gratifying that scientists have developed albumin nanoparticles as a novel delivery system, which can be directly injected into the marrow cavity of the skull through a minimally invasive approach and migrate along the cranial-meningeal microchannels to the central nervous system, reaching the lesion sites in the brain tissue. The results of the first application of this drug delivery technology in humans show that patients with cerebral infarction perform well in terms of postoperative tolerance, safety, and neurological function recovery, providing a preliminary basis for the clinical transformation of this strategy [189]. The successful application of this delivery system brings hope for achieving targeted regulation of E2F1 in VaD brain tissue. Although there is still a large amount of research work to be explored, E2F1 becoming a pharmacological therapeutic target for VaD is a very promising exploration direction of great significance for the development of VaD disease treatment.

## Figures and Tables

**Table 1:** Mechanisms of excitotoxicity induced by chronic cerebral hypoperfusion.

Cell/Structure Involved	Main Mechanism	Outcome/Effect	References
Presynaptic neuron	Ischemia/hypoxia → sustained glutamate release	Overactivation of postsynaptic NMDAR and other receptors → massive Ca^2+^ influx → calcium overload → neuronal death	[31]
Postsynaptic neuron	Overactivation of NMDAR and other excitatory amino acid receptors	Massive Ca^2+^ influx → calcium overload → neuronal death	[31]
Endothelial cell	Sustained activation of glutamate receptors → massive Ca^2+^ entry into endothelial cells	Mitochondrial dysfunction, ROS generation, metabolic damage → disruption of blood–brain barrier integrity → neurotoxic injury	[32,33]
Astrocyte	Glutamate transporters (energy-dependent, Na^+^ gradient-driven) take up extracellular glutamate and convert it to glutamine for recycling	Ischemia/hypoxia → energy deficit + increased intracellular Na^+^ → reverse transport of glutamate to extracellular space → glutamate accumulation → exacerbation of excitotoxicity	[34,35,36]
Microglia	Activated microglia import cystine and export glutamate via system xc^−^; release factors impair glutamate transporter function in other cells	Further extracellular glutamate accumulation → aggravated excitotoxic damage	[37]

**Table 2:** The role of BBB in the pathogenesis of VaD.

Components	Normal Structure & Function	Pathological Changes in VaD	Main Consequences	References
BBB as a whole	Composed of brain microvascular endothelial cells (BMECs), astrocyte endfeet, pericytes, and basement membrane (BM); strictly restricts harmful substances from entering the brain parenchyma; regulates exchange of nutrients and metabolic waste; maintains ion and neurotransmitter homeostasis	Chronic cerebral hypoperfusion/ischemia–reperfusion injury → BBB cellular injury or pro-inflammatory changes → BBB disruption	Increased BBB permeability → blood-derived toxins enter brain → activation of glia and neurons → oxidative stress and cell death → cognitive impairment; forms a “vicious cycle”	[80,81,89]
BMECs	Express tight junction proteins (claudin-5, occludin, ZO-1) sealing paracellular pathways; lack fenestrations and have very low pinocytosis; highly express efflux transporters; contain GLUT1 and other active transporters	mitochondrial content ↓, pinocytotic vesicles ↑, GLUT1 expression ↓	Insufficient cerebral energy supply	[56,84]
Pericytes	Embedded between BMECs and astrocytes; regulate endothelial function via “peg-socket” contacts; key for BBB formation and stability	Significant ↓ in number; loss of vascular stability; white matter microcirculatory dysfunction (fibrinogen deposition, ↓ blood flow, myelin loss); soluble marker PDGFRβ ↑	Cognitive impairment and white matter damage	[82,85,86]
Astrocyte endfeet	Wrap around vessels; secrete laminin and other paracrine factors to maintain BBB integrity; participate in interstitial fluid clearance (via AQP4)	Activation → ↓ vascular coverage; ↑ GFAP, ↓ AQP4; impaired clearance capacity; ↑ LCN2	Hippocampal neuronal death; exacerbation of neuroinflammation and metabolic disturbance	[83,87,88]

↑ indicates increase; **↓** indicates decrease.

## Data Availability

No new data were created or analyzed in this study. Data sharing is not applicable to this article.
